# Supplementary material for: The Preventive Role of mRNA Vaccines in Reducing Death among Moderate Omicron-Infected Patients: A Follow-Up Study
Source: Viruses. 2022 Nov 24;14(12):2622. doi: 10.3390/v14122622 (PMC9783377; doi:10.3390/v14122622)

viruses

MDPI

Article

The Potential Therapeutic Effect of mRNA Vaccine on Better Survival of Moderate Omicron-infected Patients: A Follow-Up Study

Supplementary Materials

Table S1. Estimated regression coefficients with consideration of interaction term of vaccination and the severity of disease with adjustment for age.

Variable	Regression coefficient	Standard error	Chi-square	p-value
**Age**	0.0109	0.0018	38.55	<0.0001
**Severity**				
**Moderate**	(Reference)			
**Severe**	2.6021	0.0893	848.6	<0.0001
**Vaccination**				
**Unvaccinated**	(Reference)			
**Vaccinated**	-0.2913	0.1156	6.35	0.0118
**Interaction**				
**Severity* Vaccinated**	0.2929	0.1305	5.04	0.0248

Copyright: © 2022 by the authors. Licensee MDPI, Basel, Switzerland. This article is an open access article distributed under the terms and conditions of the Creative Commons Attribution (CC BY) license (https://creativecommons.org/licenses/by/4.0/).

Table S2. Estimated regression coefficients with consideration of interaction term of vaccination split into primary two-dose and booster group and the severity of disease with adjustment for age.

Variable	Regression coefficient	Standard error	Chi-square	p-value
**Age**	0.0109	0.0018	38.53	<0.0001
**Severity**				
**Moderate**	(Reference)			
**Severe**	2.6086	0.0895	848.7	<0.0001
**Vaccination**				
**Unvaccinated**	(Reference)			
**Primary 2-dose group**	-0.1401	0.1694	0.68	0.4081
**Booster group**	-0.3467	0.1295	7.16	0.0074
**Interaction**				
**Severity*primary 2-dose**	0.0400	0.1913	0.0436	0.8345
**Severity*Booster**	0.3989	0.1461	7.4521	0.0063

Figure S1. Survival curve of hospitalized patients of primary 2-dose, booster vaccine and unvaccinated group.

(A) Moderate patients

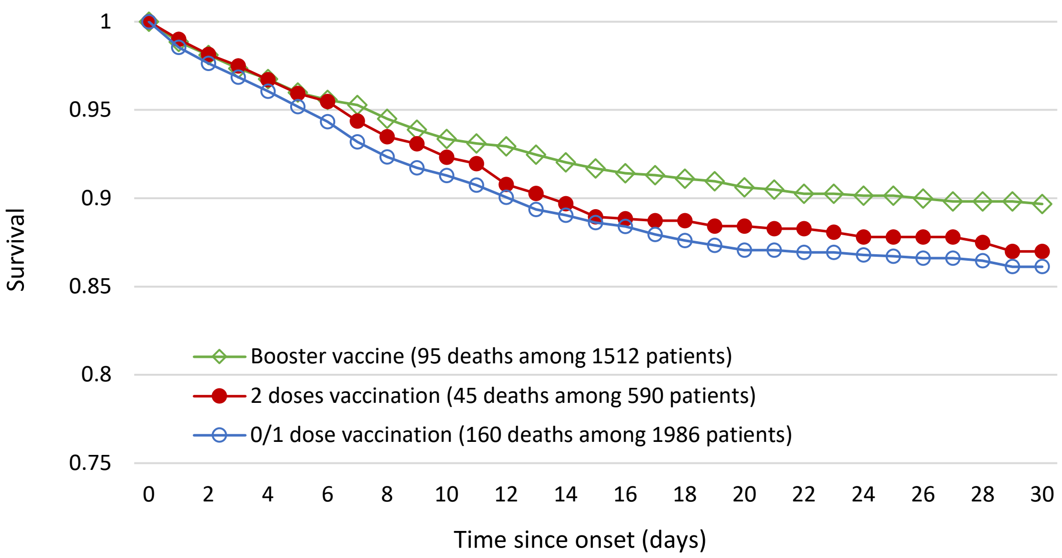

(B) Severe patients

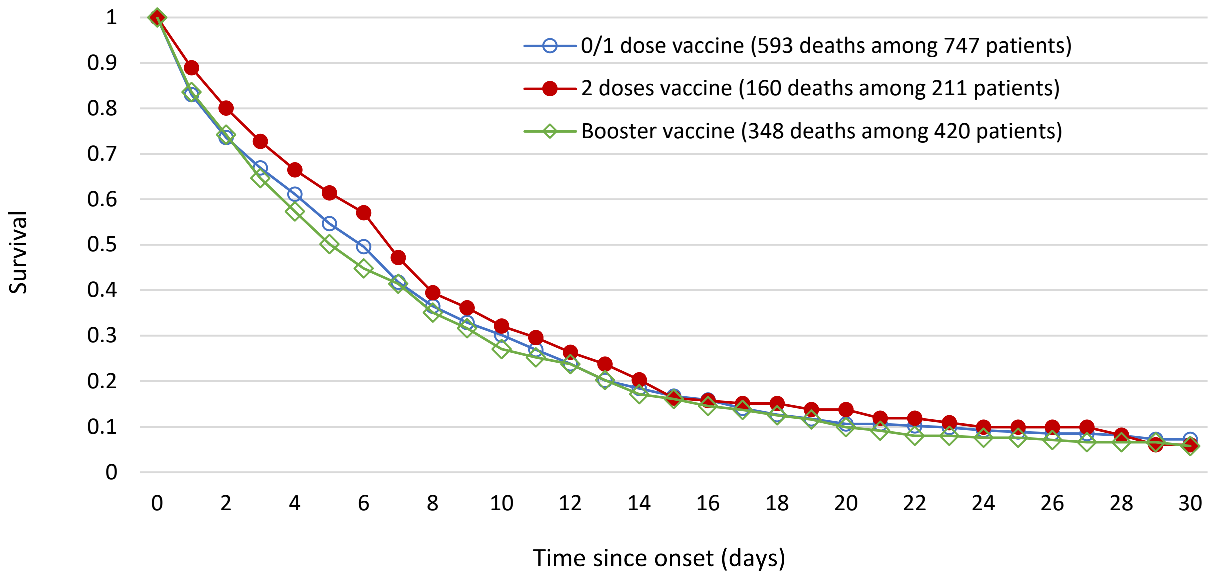

Supplement: Supplementary file 1 [file viruses-14-02622-s001.zip › viruses-2046990-supplementary.pdf]
